# Diagnostic value of susceptibility-based imaging and apparent diffusion coefficient for differentiating multiple system atrophy-Parkinsonian type from Parkinson's disease

**DOI:** 10.3389/fnagi.2026.1967702

**Published:** 2026-10-14

**Authors:** Byeongpil Moon, So Yeon Won, Jonghyo Youn, Yujin Seo, Jinyoung Youn, Eung Yeop Kim, Jongho Lee, Beomseok Sohn

**Affiliations:** 1 Department of Electrical and Computer Engineering, Seoul National University, Seoul, Republic of Korea; 2 Department of Radiology, Samsung Medical Center, Sungkyunkwan University School of Medicine, Seoul, Republic of Korea; 3 Department of Neurology, Samsung Medical Center, Sungkyunkwan University School of Medicine, Seoul, Republic of Korea

**Keywords:** apparent diffusion coefficient, multiple system atrophy-Parkinsonian type, Parkinson's disease, susceptibility-weighted imaging, χ-separation-derived paramagnetic susceptibility map

## Abstract

**Objective:**

To characterize the diagnostic performance of susceptibility-weighted imaging (SWI), χ-separation-derived paramagnetic susceptibility (χ_para_), and apparent diffusion coefficient (ADC) for differentiating multiple system atrophy-Parkinsonian type (MSA-P) from Parkinson's disease (PD).

**Materials and methods:**

This retrospective case-control study included 27 drug-naïve patients with MSA-P and 27 age- and sex-matched drug-naïve patients with PD. Clinical diagnosis by board-certified neurologists served as the reference standard. χ_para_ maps were generated from multi-echo gradient-recalled echo images using χ-separation, and ADC maps were derived from diffusion-weighted imaging. Two blinded radiologists qualitatively assessed SWI and ADC images using prespecified putaminal criteria. Quantitative analyses included voxel-wise group comparison and receiver operating characteristic (ROC) analysis using mean region-of-interest (ROI) values. Candidate logistic-regression models were assessed using leave-one-out cross-validation.

**Results:**

Qualitative assessment yielded sensitivity (85.2%), specificity (100%) and accuracy (92.6%) for SWI; the corresponding ADC assessments were 70.4%, 77.8% and 74.1%, respectively. Voxel-wise χ_para_ analysis revealed a rim-shaped susceptibility increase in the posterior putamen, whereas ADC differences were localized to cerebellar white matter, including the middle cerebellar peduncle (MCP). In ROI-based classification, putaminal χ_para_ yielded a sensitivity of 92.6% and ADC of the MCP showed a specificity of 96.3%. An exploratory model combining putaminal χ_para_ and ADC in the MCP yielded a leave-one-out cross-validation AUC of 0.927.

**Conclusion:**

By evaluating susceptibility-based imaging and ADC in the same cohort, this study characterized their respective regional imaging features and diagnostic performance for differentiating MSA-P from PD. χ_para_ revealed an observed rim-shaped susceptibility pattern in the posterior putamen, supporting further evaluation of this pattern as a candidate imaging finding in MSA-P.

## 1 Introduction

Accurate differentiation between multiple system atrophy-Parkinsonian type (MSA-P) and Parkinson's disease (PD) remains particularly challenging in the early disease stage, where clinical features and treatment responses overlap (Poewe and Wenning, 2002). The 2022 Movement Disorder Society (MDS) diagnostic criteria for clinically established MSA require at least one brain MRI marker of atrophy or diffusivity change in the putamen or infratentorial structures (Schocke et al., 2002; Pellecchia et al., 2009; Baudrexel et al., 2014; Barbagallo et al., 2016; Wenning et al., 2022). Among these, apparent diffusion coefficient (ADC) is one of the most commonly used diffusion markers in clinical practice. These MRI markers are typically assessed through visual inspection by experienced neuroradiologists and have demonstrated high specificity for differentiating MSA from PD. However, prior studies have reported that structural and diffusion-based MRI markers, while highly specific, demonstrate limited sensitivity for differentiating MSA-P from PD (Ito et al., 2007; Chung et al., 2009; Wilkens et al., 2024). Advanced MRI approaches provide structural, diffusion, susceptibility, functional, and radiomic readouts in parkinsonian disorders; however, differences in the properties measured and their dependence on acquisition, analytical methods, and study context complicate comparisons across studies (Barbagallo et al., 2016; Ito et al., 2017; Huang et al., 2025; Vaccarino et al., 2025; Quattrocchi et al., 2026).

As an alternative, susceptibility-based imaging techniques such as susceptibility-weighted imaging (SWI) and quantitative susceptibility mapping (QSM) have been highlighted for their ability to detect tissue magnetic susceptibility alterations, including disease-specific iron-related effects. SWI has shown promise in detecting putaminal iron-related susceptibility changes, yielding improved diagnostic sensitivity for differentiating MSA-P from PD (Lee and Baik, 2011; Hwang et al., 2015; Meijer et al., 2015). QSM studies have similarly demonstrated elevated magnetic susceptibility in the putamen, pallidum, and substantia nigra of patients with MSA (Lancione et al., 2022; Marxreiter et al., 2023). More recently, χ-separation-derived paramagnetic susceptibility (χ_para_) mapping has enabled selective quantification of paramagnetic iron-related signals, whereas conventional QSM reflects their net susceptibility (Shin et al., 2021; Shibata et al., 2025; Yan et al., 2025). Susceptibility-based imaging (SWI, χ_para_) and ADC provide distinct biophysical contrasts related to tissue magnetic susceptibility and water diffusivity, respectively. To our knowledge, their regional features and diagnostic performance have not been evaluated within the same MSA-P and PD cohort.

This study aimed to characterize the regional features and diagnostic value of susceptibility-based imaging and ADC for differentiating MSA-P from PD. Qualitatively, radiologists' interpretation of SWI and ADC was evaluated. Quantitatively, voxel-wise spatial patterns of χ_para_ and ADC were examined. Region-of-interest (ROI)-based diagnostic performance was evaluated for χ_para_ and ADC, followed by exploratory analysis of their combined performance. *Post hoc* exploratory subgroup analyses were performed according to disease duration.

## 2 Materials and methods

### 2.1 Subjects

This single-center retrospective matched case-control study was approved by the Institutional Review Boards of Samsung Medical Center (2025-01-053-001) and Seoul National University (E2606/004-004). Both boards waived the requirement for informed consent. Between December 2021 and October 2023, eligible drug-naive patients who presented to the movement disorders clinic, underwent brain MRI, and were diagnosed with MSA-P by board-certified neurologists were retrospectively included. Age- and sex-matched patients with PD were selected from a pool of 277 eligible drug-naïve patients with PD using exact sex matching and nearest-neighbor matching on age in months, without replacement or an age caliper (Figure 1). The sample size was determined by the number of eligible MSA-P cases available during the study period. The mean absolute within-pair age difference was 0.45 ± 0.37 years. Each group comprised 27 patients, including 15 male and 12 female patients. The mean age was 63.7 ± 8.3 years in the MSA-P group and 63.5 ± 8.5 years in the PD group. PD was diagnosed according to the MDS diagnostic criteria for PD (Postuma et al., 2015), and established (*n* = 19) or probable (*n* = 8) MSA-P was determined based on the MDS diagnostic criteria for MSA (Wenning et al., 2022). The final clinical diagnosis served as the reference standard and was based on a comprehensive assessment integrating longitudinal clinical course, treatment response, autonomic dysfunction and patient clinical features, and routine MRI (T2, FLAIR, GRE, T1, contrast-enhanced T1) findings used for MDS diagnostic classification. Clinical follow-up duration, defined as the interval from diagnosis to the last outpatient visit, was 767.8 ± 568.4 days, with a median of 762 days (IQR, 214.5–1,322.5 days). All participants underwent longitudinal clinical follow-up, and no diagnosis was reclassified during follow-up. The interval between MRI acquisition and the final clinical diagnosis was 68.7 ± 99.5 days, with a median of 34 days (IQR, 9–89.5 days). The research-prepared SWI, ADC, and χ_para_ images evaluated in this study, as well as all ROI measurements and model outputs, were unavailable to the neurologists when the reference diagnoses were established. All patients in the eligible source pool had the GRE and DWI sequences required for the analyses, and no patients were excluded because of poor image quality. Supplementary material 1 provides the detailed brain MRI acquisition parameters. Demographics and clinical data, including age, sex, disease duration, and Movement Disorder Society-Unified Parkinson's Disease Rating Scale (MDS-UPDRS) Part III motor examination scores, calculated as the sum of all Part III item scores, were collected.

**Figure 1:**
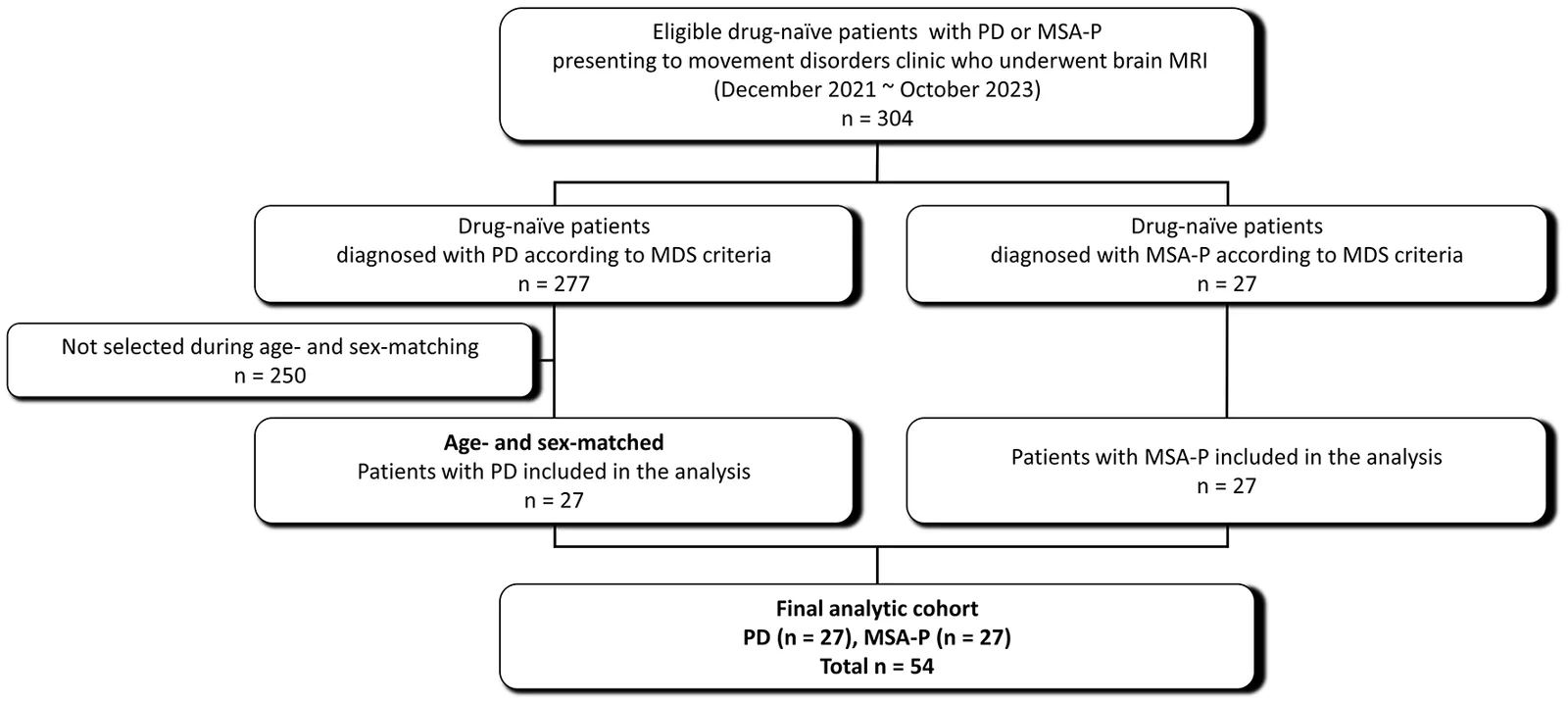
Study cohort flowchart. Flowchart showing patient retrieval and final cohort selection. PD controls were selected from patients diagnosed with PD according to Movement Disorder Society criteria and matched to the MSA-P group by age and sex. Required MRI sequences included multi-echo GRE and DWI. MSA-P, multiple system atrophy-Parkinsonian type; PD, Parkinson's disease; MDS, Movement Disorder Society; GRE, Gradient-recalled Echo; DWI, Diffusion-weighted Imaging.

### 2.2 Qualitative assessment

Two radiologists (a third-year resident and a board-certified neuroradiologist with 9 years of experience) independently reviewed the SWI and ADC images while blinded to clinical diagnosis and clinical information. SWI and ADC images were evaluated in separate reading sessions held 1 week apart under routine clinical reading conditions. Within each session, cases were presented in a randomized order irrespective of diagnosis. On SWI, a positive finding for MSA-P was defined as a continuous hypointense rim along the posterolateral putamen, with optional accompanying atrophy. On ADC, increased putaminal diffusivity involving the posterolateral putamen on at least two contiguous slices was considered positive. Patients were classified as MSA-P if either hemisphere was positive. Discrepancies were resolved by consensus.

### 2.3 Image processing

Gradient-recalled echo (GRE) phase data were unwrapped using the rapid open-source minimum spanning tree algorithm (ROMEO, mritools_win_3.2.8) (Dymerska et al., 2021) and combined using signal-to-noise-weighted averaging. Background fields were removed using V-SHARP (STISuite_v3.0) (Wu et al., 2012), yielding a local field map. R2^*^ maps were obtained by a mono-exponential fitting algorithm (Auto-Regression on Linear Operations) (Pei et al., 2015), and brain masks were extracted from the first GRE magnitude echo using the MEDI toolbox (de Rochefort et al., 2010; Liu et al., 2012). Local field and R2^*^ maps were resampled to 1 mm isotropic resolution to match the training input resolution required for QSMnet_ME_ (Yoon et al., 2018; Kim et al., 2025) and χ-sepnet (Jung et al., 2022; Kim et al., 2025). Figure 2 illustrates the image processing and analysis pipelines.

**Figure 2:**
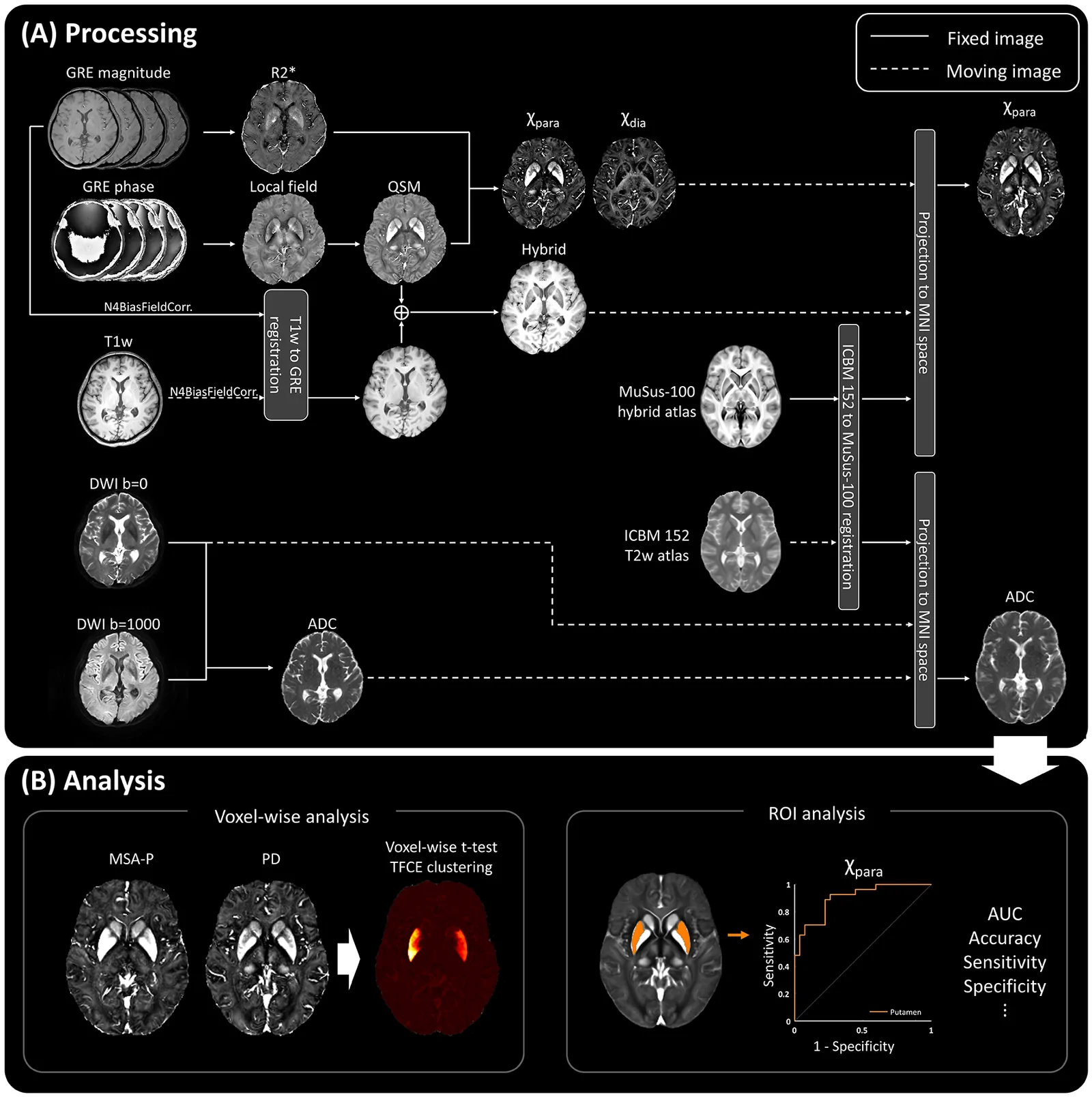
Data processing pipeline. **(A)** Processing pipeline. χ_para_ maps are computed from multi-echo GRE magnitude and phase images using χ-separation. ADC maps are estimated by fitting DWI data. A hybrid image is created and non-linearly registered to the MuSus-100 hybrid atlas; the same transformation is applied to the χ_para_ map. Separately, the DWI *b* = 0 image is registered to the ICBM T2-weighted atlas, and the resulting transformation is applied to the ADC map. Solid lines indicate fixed images, and dashed lines represent moving images. **(B)** Analysis pipeline. Voxel-wise group comparisons are performed in MNI space using TFCE with FWE correction. ROI-based diagnostic performance is subsequently evaluated. χ_para_, χ-separation-derived paramagnetic susceptibility; GRE, gradient-recalled echo; ADC, apparent diffusion coefficient; DWI, diffusion-weighted imaging; ICBM, International Consortium for Brain Mapping; MNI, Montreal Neurological Institute; TFCE, threshold-free cluster enhancement; FWE, family-wise error.

R2′ maps were estimated from the resampled R2^*^ maps using R2PRIMEnet (Kim et al., 2025). Quantitative susceptibility mapping (QSM) was generated using QSMnet_ME_ and χ_para_ and diamagnetic susceptibility (χ_dia_) maps were generated using χ-sepnet. χ_para_ maps were resampled to original resolution; all processing was performed using the χ-separation toolbox v1.2 with the pretrained R2PRIMEnet, QSMnetME, and χ-sepnet checkpoints corresponding to the publicly available network weights (Shin et al., 2021). The checkpoint files used for R2PRIMEnet, QSMnetME, and χ-sepnet were 240531_R2PRIMEnet.onnx, 240904_QSMnet.onnx, and 240904_xsepnet.onnx, respectively. None of the participants in the present study were included in the training, validation, or tuning datasets used to develop these networks. No additional input-intensity normalization was applied, and the QSMnetME output was used directly without further susceptibility referencing. Susceptibility maps were reconstructed in parts per million (ppm) and displayed in parts per billion (ppb) after multiplication by 1,000.

ADC maps were calculated by mono-exponential fitting of one reconstructed *b* = 0 volume and one directionally averaged *b* = 1000 s/mm^2^ volume; both *b* values were acquired with three signal averages. Only scanner-reconstructed *b* = 0 and directionally averaged *b* = 1000 s/mm^2^ images were available; therefore, dedicated motion, eddy-current, and susceptibility-induced EPI-distortion correction could not be performed. Non-positive signal values were replaced with machine epsilon (2.22 × 10^−16^) before logarithmic mono-exponential fitting, and voxels outside the FSL-derived brain mask were subsequently set to zero. Brain extraction was performed using FSL (Smith, 2002).

### 2.4 Image registration

All images were registered to the Montreal Neurological Institute (MNI) standard space using Advanced Normalization Tools (ANTs) (Avants et al., 2009, 2011). Mutual information was used as the similarity metric for the rigid and affine registration stages, whereas cross-correlation was used for non-linear symmetric normalization (SyN). For χ_para_ image registration, a hybrid image was employed to ensure accurate alignment of subcortical structures. The hybrid image was constructed by linearly registering the B1 bias field-corrected (N4BiasFieldCorrection) (Avants et al., 2009, 2011) T1-weighted image to the B1 bias field-corrected first echo GRE magnitude image, followed by combining the aligned T1 and QSM images. Then, the hybrid image was non-linearly registered to the MuSus-100 hybrid atlas (He et al., 2023), and the resulting transformation was applied to the χ_para_ maps using sinc interpolation.

Before ADC registration, the T2-weighted atlas from the International Consortium for Brain Mapping (ICBM) 152 Non-linear template (Fonov et al., 2009, 2011) was linearly registered to the MuSus-100 hybrid atlas to ensure consistent ROI alignment across susceptibility and diffusion maps. To improve registration precision, the DWI *b* = 0 image was resampled to 1 mm isotropic resolution using linear interpolation to reduce ringing artifact near high intensity CSF. The resampled DWI was then non-linearly registered to the T2-weighted atlas, and the resulting transformation was applied to the ADC maps.

For each participant, registration quality was visually assessed by overlaying the corresponding atlas masks on the hybrid, χ_para_, and ADC images and inspecting the alignment of the brain contour, caudate nucleus, putamen, globus pallidus, and MCP; no gross registration failure required reprocessing or exclusion.

### 2.5 Region of interest definition

Parcellation labels for the deep gray matter and thalamus were obtained from the χ-separation atlas (Min et al., 2024). Because the χ-separation atlas is inherently aligned to the MuSus-100 hybrid atlas, these labels were directly compatible with images registered to the reference space. Cerebellar white matter ROIs were extracted from the cerebellar regions of the ICBM DTI 81 atlas (Mori et al., 2008) and the corresponding white matter parcellation was segmented. To ensure accurate alignment of the cerebellar parcellation, the ICBM T1-weighted atlas was linearly registered to the MuSus-100 T1-weighted atlas, and the resulting transformation was applied to the ICBM DTI 81 cerebellar parcellation masks. To mitigate potential misregistration at the cerebellar boundaries, the resulting cerebellar ROIs were eroded by 1 mm.

### 2.6 Demographic analysis

Normality of age, disease duration, and MDS-UPDRS Part III scores was assessed using the Kolmogorov-Smirnov and Shapiro-Wilk tests. Age was normally distributed and compared using a two-sample *t*-test. Disease duration and MDS-UPDRS Part III scores violated the normality assumption and were compared using the Mann-Whitney *U*-test. Group differences in sex distribution were assessed using Pearson's chi-square test.

### 2.7 Voxel-wise comparison

Voxel-wise analyses were performed to compare spatial differences in χ_para_ and ADC between patients with MSA-P and PD. To improve the signal-to-noise ratio (SNR) and account for potential misregistration, spatial smoothing was applied using a minimal 1 mm full width at half maximum (FWHM) Gaussian kernel to preserve structural detail. For each modality, the voxel-wise general linear model included diagnostic group as the effect of interest, with mean-centered age and one-hot-encoded sex as nuisance covariates. One-tailed MSA-P > PD contrasts were specified before group-level inferential testing based on prior reports of higher putaminal susceptibility and increased putaminal or infratentorial diffusivity in MSA-P (Schocke et al., 2002; Nicoletti et al., 2006; Sjöström et al., 2017). Multiple comparisons were corrected using threshold-free cluster enhancement (TFCE) (Friston, 2003; Smith and Nichols, 2009) and maximum permutation statistics (10,000 permutations) using TFCE toolbox (version r276) of Statistical Parametric Mapping, version 12 (SPM12) (Friston, 2003). Family-wise error (FWE) corrected *p*-values were derived using a Gamma-tail approximation to the permutation distribution of the maximum TFCE statistic. Statistical significance was set at a corrected *p*-value < 0.05.

For each cluster, the number of voxels, maximum TFCE value, corresponding corrected *p*-value, and MNI coordinates of the peak voxel were reported in Supplementary Tables 1, 2. To provide anatomical context, we identified the ROI containing the peak TFCE voxel and calculated the proportion of each anatomical ROI occupied by the cluster. For detailed anatomical reporting, ROIs were separated into left and right hemispheres.

### 2.8 Region of interest-based receiver operating characteristic analysis and diagnostic performance

Receiver operating characteristic (ROC) curves and area under the curve (AUC) values were computed for each individual ROI using mean values from χ_para_ and ADC maps. For each ROI, a single subject-level value was calculated as the mean across all voxels in the combined left and right atlas masks. Diagnostic accuracy, sensitivity, specificity, positive predictive value (PPV), and negative predictive value (NPV) were calculated at the optimal diagnostic threshold defined by the highest Youden's index (Youden, 1950). AUC values with 95% confidence intervals were estimated non-parametrically. The 95% confidence intervals for accuracy, sensitivity, specificity, PPV and NPV were calculated using the Wilson score method. Additional ROI results, including diagnostic performance metrics for cases where multiple thresholds yielded the same highest Youden's index, are provided in Supplementary Figures 1, 2.

To explore the joint diagnostic performance of multiple ROI values, logistic-regression models were constructed using three candidate features selected based on the full-cohort voxel-wise and ROI findings: putaminal χ_para_, putaminal ADC, and MCP ADC. All three possible two-feature combinations and the one three-feature combination were evaluated. For each combination, one participant was excluded, the regression coefficients were estimated using the remaining 53 participants, and the fitted model was used to calculate the probability for the excluded participant. This procedure was repeated until a leave-one-out prediction had been generated for every participant. Because each participant was excluded when the corresponding regression coefficients were estimated, leave-one-out cross-validation (LOOCV) reduced optimism from estimating and evaluating the coefficients in the same participants. The 54 leave-one-out predictions were then used together to construct the ROC curve and calculate the AUC. However, feature prioritization and selection of the model for primary presentation were based on the full-cohort findings and were not repeated separately within each cross-validation training set. After all leave-one-out predictions had been generated, a single decision threshold was selected using Youden's index based on the predictions and known diagnoses of all 54 participants. Thus, threshold selection was not included within LOOCV.

### 2.9 Subgroup analysis by disease duration

In a *post hoc* exploratory analysis, patients were stratified into three subgroups based on disease duration at the time of MRI: < 1 year, 1–3 years, and ≥3 years. Disease duration was defined as the interval between the onset of motor symptoms (as reported by the patient or caregiver) and the MRI examination date. For each subgroup, diagnostic performance metrics were calculated for qualitative SWI and ADC assessments, as well as χ_para_ and ADC-based quantitative analyses. To allow comparison of differentiation performance across disease durations, the same optimal thresholds derived from the full-cohort ROI analysis were applied to all subgroups.

### 2.10 Supplementary ROI-based analysis

To investigate regional group differences between patients with MSA-P and PD, ROI-based comparisons were performed using the atlas-defined regions described above. For each ROI, normality was assessed using the Shapiro-Wilk test. Group differences were evaluated using independent two-sample *t*-tests or Mann-Whitney *U* test. *p*-values were adjusted using the Bonferroni correction. The corresponding ROI-based results are summarized as box plots for individual ROIs in Supplementary Figures 3, 4. All data processing was performed using MATLAB (MATLAB 2022a, MathWorks Inc., Natick, MA, USA), and statistical analyses were conducted using SPSS (SPSS Statistics Version 29.0; IBM Corp., Armonk, NY, United States) and MATLAB.

## 3 Results

### 3.1 Patient demographics

Figure 1 shows the study cohort selection process. Table 1 summarizes the demographic and clinical information. Age, sex, and disease duration did not differ significantly between the MSA-P and PD groups. Disease duration averaged 24.5 ± 19.9 months for MSA-P and 23.0 ± 14.8 months for PD. Median MDS-UPDRS Part III scores were 25 [17–43] and 10 [7.0–17.5] in the MSA-P and PD groups, respectively (*p* < 0.001).

**Table 1:** Demographics of patients.

Feature	MSA-P (*n* = 27)	PD (*n* = 27)	*p*-value
Age (mean ± SD, years)	63.7 ± 8.3	63.5 ± 8.5	0.963
Sex (male/female)	15/12	15/12	1.000
Disease duration (mean ± SD, months)	24.5 ± 19.9	23.0 ± 14.8	0.758
MDS-UPDRS Part III (median [IQR])	25 [17–43] (*n* = 19)	10 [7.0–17.5] (*n* = 27)	< 0.001

SD, standard deviation; IQR, Interquartile Range; MDS-UPDRS, Movement Disorder Society-Unified Parkinson's Disease Rating Scale; MSA-P, multiple system atrophy-Parkinsonian type; PD, Parkinson's disease.

### 3.2 Voxel-wise analysis on χ-separation-derived paramagnetic susceptibility and apparent diffusion coefficient

Group average and group difference maps of χ_para_ demonstrated elevated susceptibility in the posterior putamen and globus pallidus in the MSA-P group. The resulting TFCE clusters identified regions with significant differences between the groups. Notably, χ_para_ clusters exhibited a rim-shaped pattern along the posterior putamen (Figure 3). Supplementary Tables 1, 2 summarize the cluster distribution. The two largest χ_para_ clusters had their peak TFCE voxels within the right and left putaminal ROIs, overlapping the putamen by 61% and 63%, respectively.

**Figure 3:**
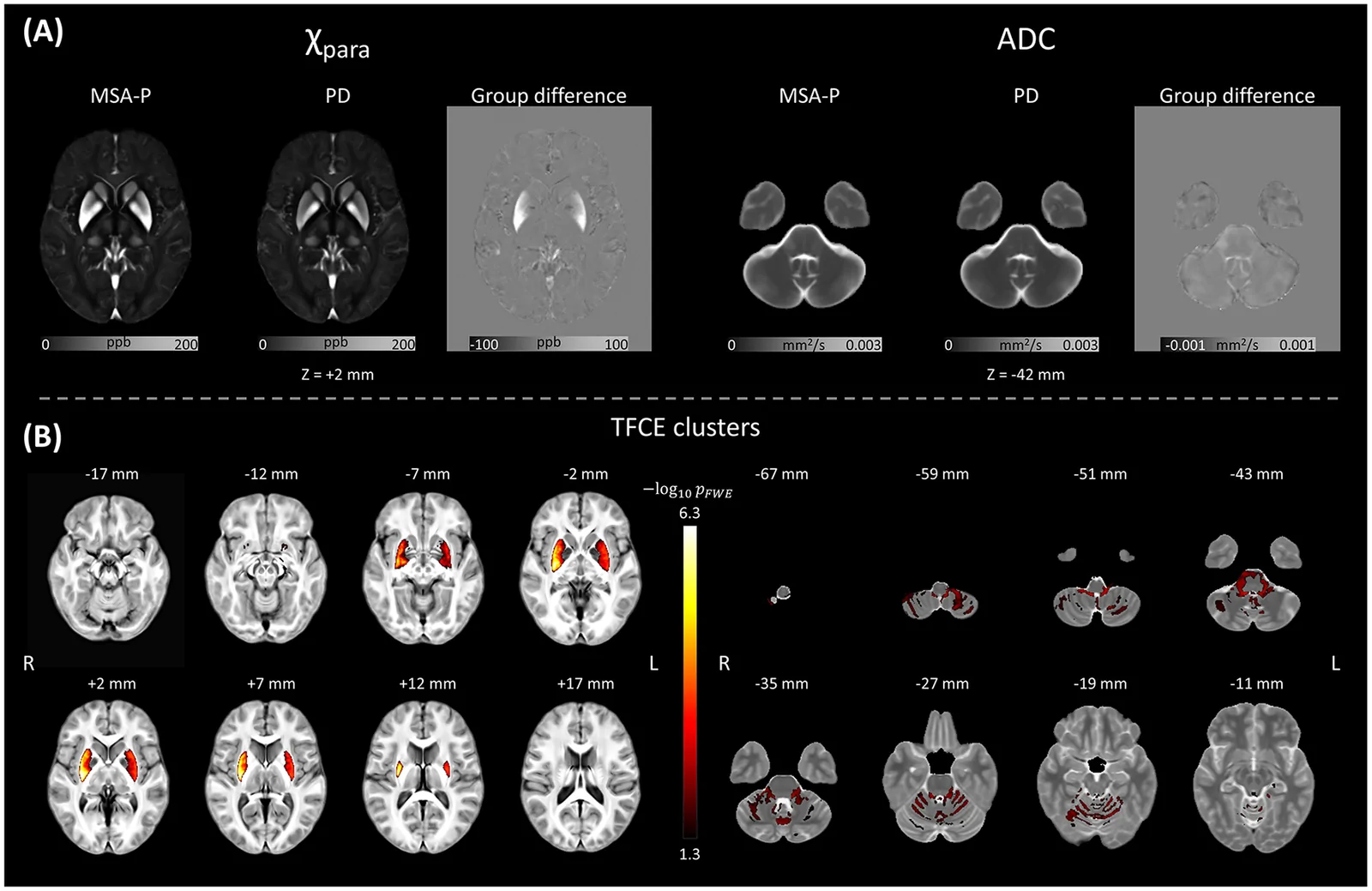
Voxel-wise analysis results. **(A)** Group-average and group-difference maps of χ_para_ and ADC. χ_para_ demonstrates a rim-shaped increase in the posterior putamen in MSA-P compared to PD. In contrast, group differences in ADC are localized to the cerebellar white matter, including the MCP. **(B)** TFCE-derived clusters (FWE-corrected *p* < 0.05). Clusters are displayed at 5-mm slice intervals for χ_para_ and 8-mm intervals for ADC. Within each cluster, corrected voxel-wise *p*-values are log-transformed and visualized using –log10(p_FWE_), ranging from 1.3 (corresponding to *p* = 0.05) to a maximum of 6.3. The resulting color-coded maps are overlaid on the hybrid atlas for χ_para_ and the T2-weighted atlas for ADC. MSA-P, multiple system atrophy-Parkinsonian type; PD, Parkinson's disease; χ_para_, χ-separation-derived paramagnetic susceptibility; TFCE, threshold-free cluster enhancement; FWE, family-wise error; ADC, apparent diffusion coefficient; MCP, middle cerebellar peduncle.

The ADC group-difference map showed clusters primarily in the cerebellar white matter, and the MCP showed the largest overlap among predefined ROIs.

### 3.3 Qualitative diagnostic performance

SWI-based qualitative assessment demonstrated sensitivity, specificity, and accuracy of 85.2%, 100%, and 92.6%, respectively (Table 2). ADC-based qualitative assessment yielded sensitivity, specificity, and accuracy of 70.4%, 77.8%, and 74.1%, respectively. The agreement between radiologists was almost perfect, with κ = 0.962 (95% CI, 0.877–1.000) for SWI and κ = 0.963 (95% CI, 0.882–1.000) for ADC. Reader-specific pre-consensus contingency tables and corresponding sensitivity and specificity estimates with 95% confidence intervals are provided in Supplementary Table 3. Figure 4 illustrates the representative MSA-P and PD cases.

**Table 2:** Qualitative and quantitative diagnostic performance for differentiating MSA-P from PD.

Analysis type	Imaging feature (ROI)		AUC	Accuracy	Sensitivity	Specificity	PPV	NPV
Qualitative	SWI		N/A	0.926 (50/54) (CI: 0.824–0.971)	0.852 (23/27) (CI: 0.675–0.941)	1.000 (27/27) (CI: 0.875–1.000)	1.000 (23/23) (CI: 0.857–1.000)	0.871(27/31) (CI: 0.711–0.949)
	ADC		N/A	0.741 (40/54) (CI: 0.611–0.839)	0.704 (19/27) (CI: 0.515–0.841)	0.778 (21/27) (CI: 0.592–0.894)	0.760 (19/25) (CI: 0.566–0.885)	0.724 (21/29) (CI: 0.543–0.853)
Quantitative	χ_para_	Put	0.900 (CI: 0.821–0.979)	0.833 (45/54) (CI: 0.713–0.910)	0.926 (25/27) (CI: 0.766–0.979)	0.741 (20/27) (CI: 0.553–0.868)	0.781 (25/32) (CI: 0.612–0.890)	0.909 (20/22) (CI: 0.722–0.975)
	ADC	MCP	0.846 (CI: 0.734–0.959)	0.833 (45/54) (CI: 0.713–0.910)	0.704 (19/27) (CI: 0.515–0.841)	0.963 (26/27) (CI: 0.817–0.993)	0.950 (19/20) (CI: 0.764–0.991)	0.765 (26/34) (CI: 0.600–0.876)
		Put	0.675 (CI: 0.529-0.821)	0.685 (37/54) (CI: 0.553–0.793)	0.444 (12/27) (CI: 0.276–0.627)	0.926 (25/27) (CI: 0.766–0.979)	0.857 (12/14) (CI: 0.601–0.960)	0.625 (25/40) (CI: 0.470–0.758)
	Combined	χ_para_ (Put) and ADC (MCP)	0.927 (CI: 0.853–1.000)	0.889 (48/54) (CI: 0.778–0.948)	0.852 (23/27) (CI: 0.675–0.941)	0.926 (25/27) (CI: 0.766–0.979)	0.920 (23/25) (CI: 0.750–0.978)	0.862 (25/29) (CI: 0.694–0.945)

AUC, accuracy, sensitivity, specificity, PPV, and NPV are reported for both qualitative (radiologists' assessment) and quantitative (ROI-based classification) diagnostic evaluations, along with classification proportions and 95% confidence intervals. AUC could not be computed for the qualitative analysis due to its categorical output. In the quantitative analysis, χ_para_ in the putamen yielded two thresholds with identical Youden's index; the threshold with the higher sensitivity is reported. AUC, Area under the curve; PPV, Positive predictive value; NPV, Negative predictive value; SWI, Susceptibility-weighted imaging; ADC, Apparent diffusion coefficient; Put, Putamen; MCP, Middle cerebellar peduncle.

**Figure 4:**
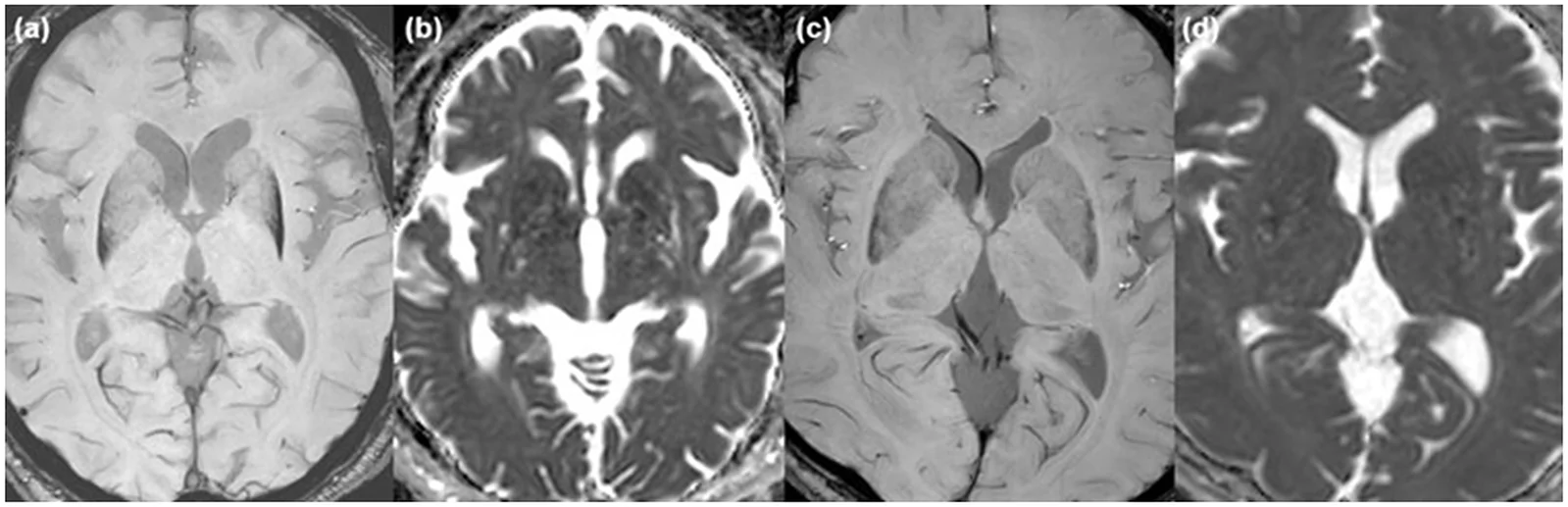
Representative MSA-P and PD cases. **(a, b)** A 65-year-old male patient with MSA-P (disease duration 24 months, left two panels) shows putaminal atrophy with a hypointense rim on SWI and increased diffusivity in the left putamen on ADC maps. **(c, d)** In contrast, an age- and disease duration-matched PD patient (right two panels) demonstrates no putaminal changes typical of MSA-P. MSA-P, multiple system atrophy-Parkinsonian type; PD, Parkinson's disease; SWI, susceptibility-weighted imaging; ADC, apparent diffusion coefficient.

### 3.4 Quantitative diagnostic performance (region-of-interest-based)

In the ROI-based analysis (Table 2, Figure 5), putaminal χ_para_ yielded an AUC of 0.900 (95% CI: 0.821–0.979), sensitivity of 92.6%, and specificity of 74.1%. ADC in the MCP showed an AUC of 0.846 (95% CI: 0.734–0.959), sensitivity of 70.4%, and specificity of 96.3%. Putaminal ADC yielded an AUC of 0.675 (95% CI: 0.529–0.821). Additional ROI results are provided in Supplementary Figures 1, 2. In sensitivity analyses, adjustment for disease duration minimally changed the diagnostic-group coefficients for putaminal χ_para_ and MCP ADC, and disease duration was not independently associated with either measure (Supplementary material 2).

**Figure 5:**
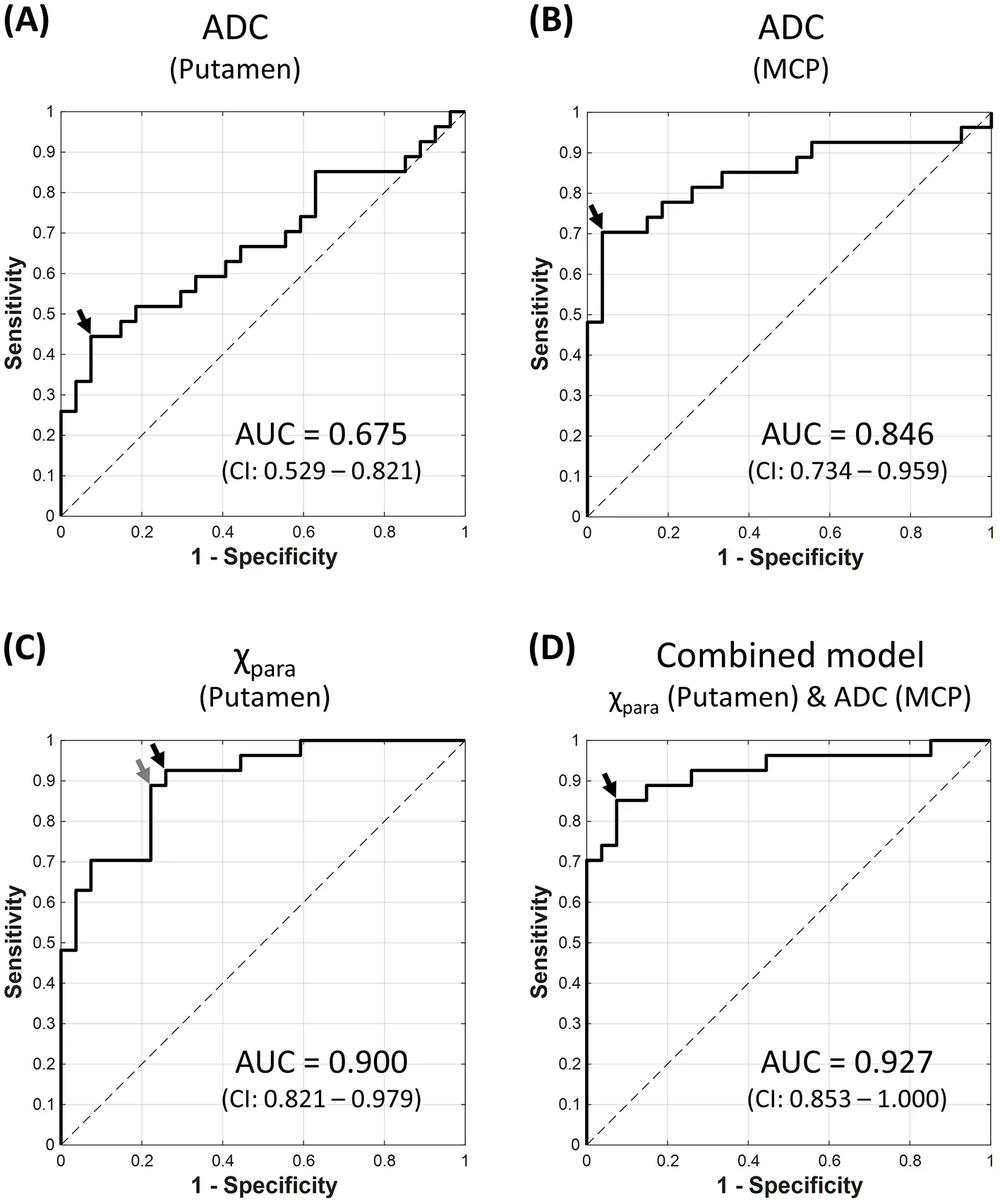
ROC curves and AUC values. **(A, B)** ROC curves for ADC in the putamen and MCP. AUC values with 95% confidence intervals are shown. Black arrows indicate optimal thresholds based on Youden's index. **(C)** ROC curve for putaminal χ_para_. Two thresholds yielded the same Youden's index; the threshold with higher sensitivity (black arrow) was selected. The other threshold (gray arrow) is reported in Supplementary Figure 1. **(D)** ROC curve for the combined model (putaminal χ_para_ and ADC in MCP) based on cross-validated logistic regression probabilities. ROC, receiver operating characteristic; AUC, area under the receiver operating characteristic curve; ADC, apparent diffusion coefficient; MCP, middle cerebellar peduncle; χ_para_, χ-separation-derived paramagnetic susceptibility.

For the putaminal χ_para_-MCP ADC model, LOOCV yielded an exploratory AUC of 0.927 (95% CI: 0.853–1.000). At the Youden threshold described in the Methods, accuracy was 88.9%, sensitivity was 85.2% and specificity was 92.6%. Results for all four candidate combinations are provided in Supplementary Figure 5.

### 3.5 Diagnostic performance by disease duration

In the *post hoc* exploratory disease-duration analysis, SWI-based qualitative assessment had a specificity of 100% across all disease duration subgroups, and an accuracy of 96.6% in the 1–3-year group (Table 3). In the quantitative analysis, putaminal χ_para_ classified 15 of 15 patients in the < 1-year subgroup; sensitivity was 92.3% and 83.3%, and specificity was 68.8% and 50.0% in the 1–3-year and ≥3-year subgroups, respectively. For ADC in the MCP, specificity was 85.7%, 100%, and 100%, and sensitivity was 87.5%, 61.5%, and 66.7% across the < 1-year, 1–3-year, and ≥3-year subgroups, respectively. Detailed confidence intervals are provided in Supplementary Table 4.

**Table 3:** Diagnostic performance stratified by disease duration.

Analysis type	Imaging feature (ROI)		Duration<1 (MSA-P: PD = 8: 7)			1 ≤ Duration<3 (MSA-P: PD = 13: 16)			3 ≤ Duration (MSA-P: PD = 6: 4)		
			Acc	Sen	Spe	Acc	Sen	Spe	Acc	Sen	Spe
Qualitative	SWI		0.933 (14/15)	0.875 (7/8)	1.000 (7/7)	0.966 (28/29)	0.923 (12/13)	1.000 (16/16)	0.800 (8/10)	0.667 (4/6)	1.000 (4/4)
	ADC		0.733 (11/15)	0.625 (5/8)	0.857 (6/7)	0.759 (22/29)	0.769 (10/13)	0.750 (12/16)	0.700 (7/10)	0.667 (4/6)	0.750 (3/4)
Quantitative	χ_para_	Put	1.000 (15/15)	1.000 (8/8)	1.000 (7/7)	0.793 (23/29)	0.923 (12/13)	0.688 (11/16)	0.700 (7/10)	0.833 (5/6)	0.500 (2/4)
	ADC	MCP	0.867 (13/15)	0.875 (7/8)	0.857 (6/7)	0.828 (24/29)	0.615 (8/13)	1.000 (16/16)	0.800 (8/10)	0.667 (4/6)	1.000 (4/4)
		Put	0.600 (9/15)	0.250 (2/8)	1.000 (7/7)	0.690 (20/29)	0.385 (5/13)	0.938 (15/16)	0.800 (8/10)	0.833 (5/6)	0.750 (3/4)

Patients were categorized into three subgroups based on disease duration: < 1 year (MSA-P = 8, PD = 7), 1–3 years (MSA-P = 13, PD = 16), and ≥3 years (MSA-P = 6, PD = 4). To evaluate diagnostic performance across these subgroups, the same threshold defined by the highest Youden's index in the full cohort was applied uniformly. For χ_para_ in the putamen, threshold with the higher sensitivity was used for subgroup analysis. MSA-P, multiple system atrophy-Parkinsonian type; PD, Parkinson's disease; χ_para_, χ-separation–derived paramagnetic susceptibility; Acc, accuracy; Sen, sensitivity; Spe, specificity; ADC, apparent diffusion coefficient; MCP, middle cerebellar peduncle; Put, putamen.

## 4 Discussion

In this study, susceptibility-based imaging and ADC were evaluated within the same drug-naive, age- and sex-matched MSA-P and PD cohort using blinded qualitative assessment and quantitative analyses. Quantitatively, putaminal χ_para_ and MCP ADC showed different diagnostic characteristics, with numerically higher sensitivity and AUC (0.900) for putaminal χ_para_ and numerically higher specificity for MCP ADC in this cohort. The exploratory combined model yielded an AUC of 0.927, although its incremental performance over the individual measures was not statistically evaluated. In addition to these diagnostic findings, χ_para_ revealed an observed rim-shaped susceptibility increase in the posterolateral putamen. Its specificity and biological basis require further validation.

To our knowledge, this is the first study to delineate the spatial pattern of χ_para_ alterations in MSA-P compared to PD using χ-separation. Voxel-wise analysis revealed a rim-shaped χ_para_ increase along the posterior putamen in MSA-P, aligning with the known distribution of striatonigral degeneration (Wenning et al., 1997). Previous studies have reported increased putaminal susceptibility in MSA (Ito et al., 2017; Sjöström et al., 2017; Li et al., 2025), but QSM reflects net susceptibility and does not separate paramagnetic from diamagnetic susceptibility contributions. A prior χ-separation study reported susceptibility differences between MSA and PD (Yan et al., 2025) but did not characterize their specific pattern or analyze the MSA-P subtype separately. The increased χ_para_ may reflect iron-related tissue alterations, consistent with neuropathological findings of ferritin/Fe^3+^ deposition in the posterolateral putamen of patients with MSA-P (Matsusue et al., 2008). To assess registration accuracy and ROI alignment, visual quality control was performed for every participant, with representative native- and standard-space χ_para_ maps and registered χ_para_ and ADC maps with ROI overlays shown in Supplementary Figures 6, 7. These quality-control assessments supported registration accuracy and ROI alignment, but the contribution of putaminal atrophy, partial-volume averaging, interpolation, or tissue-composition differences to the observed rim-shaped pattern was not specifically quantified and remains a source of residual uncertainty.

Under the prespecified putaminal criteria, radiologists' SWI interpretation yielded numerically higher sensitivity and specificity than ADC in this cohort, with 100% specificity and no false positives for PD. This likely reflects the assessment of SWI hypointensity, which was visually conspicuous on SWI. In contrast, ADC-based assessment produced false positives, often from enlarged perivascular spaces or lacunar infarctions that mimic pathological diffusivity in elderly patients (Supplementary Figure 8). These findings suggest that ADC should be interpreted alongside structural or susceptibility-based MRI to avoid misdiagnosis.

Putaminal ADC showed numerically lower AUC than MCP ADC, consistent with prior studies (Nicoletti et al., 2006; Chung et al., 2009; Pasquini et al., 2022) and our voxel-wise results where the largest ADC cluster was located in the cerebellar white matter encompassing the MCP.

In the subgroup with disease duration < 1 year, putaminal χ_para_ correctly identified all 8 MSA-P cases. While this finding is noteworthy given the diminished MRI-based sensitivity typically reported in early disease (Wilkens et al., 2024), the limited subgroup size (*n* = 15), and use of a threshold derived from a cohort that included the same participants limit its interpretation as independent evidence of early diagnostic performance. For patients with a disease duration of ≥1 year, SWI-based qualitative assessment attained high specificity and accuracy. In contrast, the reduced specificity of ROI-based χ_para_ classification may reflect overlap in mean putaminal susceptibility values between groups, because a single whole-putamen threshold does not incorporate the posterolateral spatial pattern used in visual SWI assessment.

Several limitations of this study warrant discussion. First, the clinical DWI protocol used relatively thick slices with interslice gaps and yielded directionally averaged images. Dedicated correction for motion, eddy-current, and susceptibility-induced EPI-distortion was not performed. Together, these limitations may increase partial-volume and geometric-distortion effects, particularly in voxel-wise analyses and MCP measurements. However, the slice thickness reflects the standard clinical acquisition protocol; thus, the qualitative assessment represents practical diagnostic performance. The DWI protocol heterogeneity was not harmonized, and no exclusion sensitivity analysis was performed. Second, the single-center matched case-control design and relatively small study cohort (*n* = 54) may introduce selection and spectrum effects and limit statistical stability, particularly in the disease-duration subgroup analyses. Prospective validation in larger multi-center cohorts with clinically representative patients is necessary to confirm the generalizability of our findings, and subgroup analysis should therefore be interpreted cautiously. Third, routine MRI contributed to the MDS diagnostic classification, particularly for the 19 patients with clinically established MSA-P. Although the research-prepared SWI, ADC, and χ_para_ results were unavailable to the diagnosing neurologists, related routine MRI findings may have influenced the reference diagnosis. Because marker-specific diagnostic records and independent blinded re-adjudication were unavailable, incorporation bias cannot be excluded, and the diagnostic-performance estimates may be optimistic. Fourth, qualitative evaluation was restricted to the putamen and did not include infratentorial ADC changes such as MCP involvement, which may limit the scope of qualitative comparison. Fifth, because the 1:1 matched design fixed the disease prevalence at 50%, the PPV and NPV are specific to this sample composition and should not be generalized to routine clinical populations. Sixth, MDS-UPDRS Part III scores were higher in the MSA-P group but were unavailable for eight patients with MSA-P, limiting reliable motor-severity-adjusted analyses. Consequently, residual confounding by motor severity and broader clinical disease burden cannot be excluded, although greater motor impairment at a comparable disease duration may also be consistent with the rapid progression recognized as a supportive clinical feature of MSA (Wenning et al., 2022). Finally, optimal thresholds for the individual ROI analyses were derived within the full dataset using Youden's index. Therefore, the corresponding threshold-dependent metrics should be interpreted as cohort-specific estimates rather than as estimates generalizable to other datasets. For the combined models, LOOCV reduced optimism in estimating the coefficients of each fixed model; however, feature prioritization and full-cohort Youden-threshold selection were not performed within the cross-validation folds. Accordingly, the combined-model AUC and its 95% confidence interval are exploratory estimates conditional on the selected features, and its threshold-dependent metrics may remain optimistic. In addition, comparisons with other diffusion metrics such as fractional anisotropy and mean kurtosis remain to be explored.

In conclusion, this study evaluated the diagnostic characteristics of susceptibility-based imaging and ADC for differentiating MSA-P from PD within the same drug-naïve, age- and sex-matched cohort. χ_para_ revealed an observed rim-shaped susceptibility pattern in the posterior putamen, supporting further evaluation of this pattern as a candidate imaging finding in MSA-P.

## Data Availability

The datasets presented in this article are not readily available because of privacy and ethical restrictions, and access requires approval by the relevant institutional review board. Custom analysis code and deidentified ROI-level data may be made available by the corresponding author upon reasonable request, subject to approval by the relevant institutional review board. Requests to access these materials should be directed to Beomseok Sohn (beomseoksohn@gmail.com).
