# Supplementary material for: Diagnostic value of susceptibility-based imaging and apparent diffusion coefficient for differentiating multiple system atrophy-Parkinsonian type from Parkinson's disease
Source: Front Aging Neurosci. 2026 Oct 14;18:1967702. doi: 10.3389/fnagi.2026.1967702 (PMC13713838; doi:10.3389/fnagi.2026.1967702)
Supplement: Supplementary file 1 [file Data_Sheet_1.docx]

Supplementary Material

**Supplementary Material 1: MRI Imaging protocol**

MRI was performed using a 3T scanner with a 32-channel head coil (Philips Ingenia CX). Multi-echo gradient-recalled echo (GRE) images were acquired using the following scan parameters: TR = 31 ms, TE = 7.2, 13.4, 19.7, 25.9 ms, flip angle = 17°, FOV = 240 × 240 × 160 mm^3^, voxel size = 0.47 × 0.47 × 2 mm^3^. Susceptibility weighted imaging (SWI) was reconstructed online on the scanner using the vendor-provided reconstruction. Diffusion weighted images (DWI) were acquired using the following scan parameters: TR = 3020 ms, TE = 89 ms, flip angle = 90°, FOV = 240 × 240 × 143 mm^3^, voxel size = 0.94 × 0.94 × 5 mm^3^, slice gap = 30% (n = 52); voxel size = 0.94 × 0.94 × 3 mm^3^, no gap (n = 2), b-values = 0 and 1000 s/mm². The 5-mm protocol (voxel size, 0.94 × 0.94 × 5 mm³; 30% interslice gap) was used in 52 participants, whereas the 3-mm protocol (voxel size, 0.94 × 0.94 × 3 mm³; no gap) was used in one participant from each diagnostic group. One scanner-reconstructed b = 0 volume and one directionally averaged b = 1,000 s/mm² volume were used for ADC calculation; both b values were acquired with three signal averages. No dedicated motion, eddy-current, or susceptibility-induced EPI-distortion correction was performed. For the T1-weighted images, sagittal T1-weighted GRE was acquired using the following parameters: TR = 9.9 ms, TE = 4.6 ms, flip angle = 8°, FOV = 240 × 240 × 183 mm^3^, voxel size = 0.47 × 0.47 × 0.5 mm^3^. **Abbreviations:** MRI, Magnetic Resonance Imaging; TR, Repetition Time; TE, Echo Time; FOV, Field of View

**Supplementary Material 2: Disease-duration-adjusted sensitivity analyses**

To examine whether disease duration influenced the principal ROI differences, separate linear regression analyses were performed for putaminal χ_para_ and middle cerebellar peduncle (MCP) ADC. All 54 participants had complete disease-duration and ROI data. For each ROI measure, a model including diagnosis alone was compared with a model additionally adjusted for disease duration. Diagnosis was coded as 0 for PD and 1 for MSA-P, and disease duration was entered in years. Therefore, the diagnosis coefficient represents the mean difference between MSA-P and PD.

Unadjusted model: ROI value = β₀ + β₁(diagnosis) + ε

Disease-duration-adjusted model: ROI value = β₀ + β₁(diagnosis) + β₂(disease duration) + ε

For putaminal χ_para_, adjustment for disease duration minimally changed the diagnosis coefficient from 0.04889 ppm (95% CI, 0.03241–0.06538; p < 0.001) to 0.04833 ppm (95% CI, 0.03207–0.06460; p < 0.001). Disease duration was not independently associated with putaminal χ_para_ (β = 0.00453 ppm/year, p = 0.114).

For MCP ADC, the diagnosis coefficient similarly changed only minimally, from 7.60 × 10⁻⁵ mm²/s (95% CI, 4.54 × 10⁻⁵–1.07 × 10⁻⁴; p < 0.001) to 7.65 × 10⁻⁵ mm²/s (95% CI, 4.57 × 10⁻⁵–1.07 × 10⁻⁴; p < 0.001) after adjustment. Disease duration was not independently associated with MCP ADC (β = −4.20 × 10⁻⁶ mm²/s/year, p = 0.434).

Thus, adjustment for disease duration had minimal effects on the observed group differences. This analysis does not account for possible confounding by motor severity or other measures of overall disease burden.

**Supplementary Table 1. Anatomical projection of TFCE clusters in χ_para_**

|  |  |  |  | **Peak TFCE voxel coordinate** | | | |
| --- | --- | --- | --- | --- | --- | --- | --- |
| **Anatomical structure** | **Cluster size** | **Peak TFCE value** | **Peak voxel p-value (TFCE FWE)** | **X** | **Y** | **Z** | **ROI** |
| **Putamen R (61%)**  **GP R (15%)** | 7438 | 4017 | <0.001 | 32 | -15 | 2 | Putamen R |
| **Putamen L (63%)**  **GP L (14%)** | 7153 | 1895 | <0.001 | -32 | -4 | 1 | Putamen L |
|  | 36 | 486 | 0.024 | -25 | -91 | -23 | – |

Each row represents a TFCE cluster. For each anatomical structure, the percentage indicates the ROI volume relative to the total cluster volume. The peak TFCE value represents the maximum TFCE score observed within each cluster, with the corresponding family-wise error (FWE)-corrected *p*-values and MNI coordinates also reported. Characteristics of TFCE clusters derived from χ_para_-analysis. In the two largest clusters, the peak voxels with the highest TFCE values were in the right and left putamen in the MNI coordinates. **Abbreviations:** TFCE, Threshold-Free Cluster Enhancement; χ_para_, χ-separation–derived paramagnetic susceptibility; ROI, Region of Interest; MNI, Montreal Neurological Institute

**Supplementary Table 2. Anatomical projection of TFCE clusters in ADC maps**

|  |  |  |  | **Peak TFCE voxel coordinate** | | | |
| --- | --- | --- | --- | --- | --- | --- | --- |
| **Anatomical structure** | **Cluster size** | **Peak TFCE value** | **Peak voxel p-value (TFCE FWE)** | **X** | **Y** | **Z** | **ROI** |
| **MCP (9%), SCP (0.8%), ICP (0.3%)** | 36948 | 2366 | 0.001 | 9 | -45 | -43 | – |
|  | 736 | 1336 | 0.038 | -25 | -68 | -30 | – |
|  | 187 | 1286 | 0.045 | -20 | -88 | -38 | – |
|  | 101 | 1270 | 0.047 | -30 | -70 | -41 | – |
|  | 44 | 1275 | 0.046 | -24 | -25 | -11 | – |
|  | 31 | 1255 | 0.049 | -26 | -76 | -39 | – |
|  | 2 | 1253 | 0.0498 | -16 | -27 | -15 | – |

Each row represents a TFCE cluster. For each anatomical structure, the percentage indicates the ROI volume relative to the total cluster volume. The peak TFCE value represents the maximum TFCE score observed within each cluster, with the corresponding family-wise error (FWE)-corrected *p*-values and MNI coordinates also reported. Characteristics of TFCE clusters derived from ADC analysis. Large clusters were also observed in the cerebellum, with the middle cerebellar peduncle (MCP) accounting for the largest proportion of the cluster volume. The peak voxel with the highest TFCE value was located near the inferior cerebellar peduncle (ICP), but outside the predefined ROIs. **Abbreviations:** TFCE, Threshold-Free Cluster Enhancement; ADC, Apparent Diffusion Coefficient; ROI, Region of Interest; MNI, Montreal Neurological Institute

**Supplementary Table 3. Reader-specific and consensus classification results for qualitative SWI and ADC assessments**

| Reader 1: SWI | MSA-P (n=27) | PD (n=27) |
| --- | --- | --- |
| Positive | 23 | 0 |
| Negative | 4 | 27 |
| Sensitivity: 85.2 [67.5–94.1], Specificity: 100.0 [87.5–100.0] | | |

| Reader 1: ADC | MSA-P (n=27) | PD (n=27) |
| --- | --- | --- |
| Positive | 19 | 6 |
| Negative | 8 | 21 |
| Sensitivity: 70.4 [51.5–84.1], Specificity: 77.8 [59.2–89.4] | | |

| Reader 2: SWI | MSA-P (n=27) | PD (n=27) |
| --- | --- | --- |
| Positive | 22 | 0 |
| Negative | 5 | 27 |
| Sensitivity: 81.5 [63.3–91.8], Specificity: 100.0 [87.5–100.0] | | |

| Reader 2: ADC | MSA-P (n=27) | PD (n=27) |
| --- | --- | --- |
| Positive | 20 | 6 |
| Negative | 7 | 21 |
| Sensitivity: 74.1 [55.3–86.8], Specificity: 77.8 [59.2–89.4] | | |

| Consensus: SWI | MSA-P (n=27) | PD (n=27) |
| --- | --- | --- |
| Positive | 23 | 0 |
| Negative | 4 | 27 |

| Consensus: ADC | MSA-P (n=27) | PD (n=27) |
| --- | --- | --- |
| Positive | 19 | 6 |
| Negative | 8 | 21 |

MSA-P was designated as the positive condition and PD as the negative condition. A positive qualitative assessment indicated that either hemisphere met the prespecified posterolateral putaminal criterion for MSA-P, whereas a negative assessment indicated that neither hemisphere met the criterion. Individual-reader classifications are presented before consensus. Reader-specific sensitivity and specificity are reported with 95% confidence intervals calculated using the Wilson score method. Consensus diagnostic performance estimates are reported in Table 2 of the main manuscript. Abbreviations: ADC, apparent diffusion coefficient; CI, confidence interval; MSA-P, multiple system atrophy–parkinsonian type; PD, Parkinson’s disease; SWI, susceptibility-weighted imaging.

**Supplementary Table 4. Diagnostic performance according to disease-duration subgroup**

|  | | | Duration < 1  (MSA-P : PD = 8 : 7) | | | 1 ≤ Duration < 3  (MSA-P : PD = 13 : 16) | | | 3 ≤ Duration  (MSA-P : PD = 6 : 4) | | | |
| --- | --- | --- | --- | --- | --- | --- | --- | --- | --- | --- | --- | --- |
|  |  |  | Acc | Sen | Spe | Acc | Sen | Spe | Acc | Sen | Spe |  |
| Qualitative | SWI | | 0.933 (14/15)  (0.702–0.988) | 0.875  (7/8)  (0.529–0.978) | 1.000  (7/7)  (0.646–1.000) | 0.966 (28/29) (0.828–0.994) | 0.923 (12/13)  (0.667–0.986) | 1.000 (16/16)  (0.806–1.000) | 0.800 (8/10)  (0.490–0.943) | 0.667  (4/6)  (0.300–0.903) | 1.000  (4/4)  (0.510–1.000) |  |
|  | ADC | | 0.733 (11/15) (0.480–0.891) | 0.625  (5/8)  (0.306–0.863) | 0.857  (6/7)  (0.487–0.974) | 0.759 (22/29)  (0.579–0.878) | 0.769 (10/13)  (0.497–0.918) | 0.750 (12/16)  (0.505–0.898) | 0.700 (7/10)  (0.397–0.892) | 0.667  (4/6)  (0.300–0.903) | 0.750  (3/4)  (0.301–0.954) |  |
| Quantitative | χ_para_ | Put | 1.000 (15/15)  (0.796–1.000) | 1.000  (8/8)  (0.676–1.000) | 1.000  (7/7)  (0.646–1.000) | 0.793 (23/29)  (0.616–0.902) | 0.923 (12/13)  (0.667–0.986) | 0.688 (11/16)  (0.444–0.858) | 0.700 (7/10)  (0.397–0.892) | 0.833  (5/6)  (0.436–0.970) | 0.500  (2/4) (0.150–0.850) |  |
|  | ADC | MCP | 0.867 (13/15)  (0.621–0.963) | 0.875  (7/8)  (0.529–0.978) | 0.857  (6/7)  (0.487–0.974) | 0.828 (24/29)  (0.655–0.924) | 0.615 (8/13)  (0.355–0.823) | 1.000 (16/16)  (0.806–1.000) | 0.800 (8/10)  (0.490–0.943) | 0.667  (4/6)  (0.300–0.903) | 1.000  (4/4)  (0.510–1.000) |  |
|  |  | Put | 0.600 (9/15)  (0.357–0.802) | 0.250  (2/8)  (0.071–0.591) | 1.000  (7/7)  (0.646–1.000) | 0.690 (20/29)  (0.508–0.827) | 0.385 (5/13)  (0.177–0.645) | 0.938 (15/16)  (0.717–0.989) | 0.800 (8/10)  (0.490–0.943) | 0.833  (5/6)  (0.436–0.970) | 0.750  (3/4) (0.301–0.954) |  |

Disease-duration subgroup analyses were post hoc and exploratory. The thresholds derived from the full cohort were applied to the subgroups without re-estimation. Confidence intervals were calculated using the Wilson score method.

**Supplementary Figure 1. ROC curve and AUC analysis (χ_para_)**

Receiver operating characteristic (ROC) curves and diagnostic performance metrics for 14 χ_para_-based ROIs, categorized by anatomical region: **(A)** deep gray matter, **(B)** thalamic nuclei, and **(C)** cerebellar white matter. For each ROI, the area under the curve (AUC) is shown along with the 95% confidence interval. When multiple thresholds yielded the highest Youden’s index, all corresponding sensitivity and specificity values are reported. χ_para_ thresholds are reported in ppb. **Abbreviations:** ROC, Receiver Operating Characteristic; ROI, region of interest; χ_para_, χ-separation–derived paramagnetic susceptibility; AUC, area under the curve; CI, confidence interval; Cd, caudate nucleus; Put, putamen; GP, globus pallidus; NAcc, nucleus accumbens; SN, substantia nigra; RN, red nucleus; VP, ventral pallidum; STN, subthalamic nucleus; MThal, medial thalamus; LThal, lateral thalamus; Pul, pulvinar; ICP, inferior cerebellar peduncle; MCP, middle cerebellar peduncle; SCP, superior cerebellar peduncle.

**Supplementary Figure 2. ROC curve and AUC analysis (ADC)**

Receiver operating characteristic (ROC) curves and diagnostic performance metrics for 14 ADC-based ROIs, categorized by anatomical region: **(A)** deep gray matter, **(B)** thalamic nuclei, and **(C)** cerebellar white matter. For each ROI, the area under the curve (AUC) is shown along with the 95% confidence interval. When multiple thresholds yielded the highest Youden’s index, all corresponding sensitivity and specificity values are reported. **Abbreviations:** ROI, region of interest; ADC, Apparent Diffusion Coefficient; AUC, area under the curve; CI, confidence interval; Cd, caudate nucleus; Put, putamen; GP, globus pallidus; NAcc, nucleus accumbens; SN, substantia nigra; RN, red nucleus; VP, ventral pallidum; STN, subthalamic nucleus; MThal, medial thalamus; LThal, lateral thalamus; Pul, pulvinar; ICP, inferior cerebellar peduncle; MCP, middle cerebellar peduncle; SCP, superior cerebellar peduncle.

**Supplementary Figure 3. ROI-based analysis (χ_para_)**

Box plots illustrate χ_para_ values across multiple ROIs, comparing patients with MSA-P (orange) and PD (blue). For each ROI, normality was assessed using the Shapiro–Wilk test. Based on this, either a two-sample t-test (for normally distributed data) or a Mann–Whitney U-test (for non-normal data) was conducted, with all t-tests being two-tailed. P-values were corrected for multiple comparisons using the Bonferroni method, and values exceeding 1.0 after correction were clipped at 1.0. The y-axis represents χ_para_ values in ppb, ranging from 0 to 300. For χ_para_, the Mann–Whitney U test was used for the caudate nucleus, globus pallidus, nucleus accumbens, substantia nigra, ventral pallidum, subthalamic nucleus, ICP, and MCP; independent two-sample t-tests were used for the remaining ROIs. **Abbreviations:** ROI, region of interest; MSA-P, multiple system atrophy–parkinsonian type; PD, Parkinson’s disease; χ_para_, χ-separation–derived paramagnetic susceptibility; Cd, caudate nucleus; Put, putamen; GP, globus pallidus; NAcc, nucleus accumbens; SN, substantia nigra; RN, red nucleus; VP, ventral pallidum; STN, subthalamic nucleus; MThal, medial thalamus; LThal, lateral thalamus; Pul, pulvinar; ICP, inferior cerebellar peduncle; MCP, middle cerebellar peduncle; SCP, superior cerebellar peduncle.

**Supplementary Figure 4. ROI-based analysis (ADC)**

Box plots illustrate ADC values across multiple ROIs, comparing patients with MSA-P (orange) and PD (blue). For each ROI, normality was assessed using the Shapiro–Wilk test. Based on this, either a two-sample t-test (for normally distributed data) or a Mann–Whitney U-test (for non-normal data) was conducted, with all t-tests being two-tailed. P-values were corrected for multiple comparisons using the Bonferroni method, and values exceeding 1.0 after correction were clipped at 1.0. The y-axis represents ADC values in mm^2^/s, ranging from 0 to 2×10^-3^. For ADC, the Mann–Whitney U test was used for the nucleus accumbens, red nucleus, ventral pallidum, subthalamic nucleus, and lateral thalamus; independent two-sample t-tests were used for the remaining ROIs. **Abbreviations:** ROI, region of interest; MSA-P, multiple system atrophy–parkinsonian type; PD, Parkinson’s disease; ADC, apparent diffusion coefficient; Cd, caudate nucleus; Put, putamen; GP, globus pallidus; NAcc, nucleus accumbens; SN, substantia nigra; RN, red nucleus; VP, ventral pallidum; STN, subthalamic nucleus; MThal, medial thalamus; LThal, lateral thalamus; Pul, pulvinar; ICP, inferior cerebellar peduncle; MCP, middle cerebellar peduncle; SCP, superior cerebellar peduncle.

**Supplementary Figure 5. ROC curves for candidate combined logistic regression models with leave-one-out cross-validation**

ROC curves show the apparent full-cohort performance (dashed orange) and leave-one-out cross-validated performance (LOOCV; solid blue) for four candidate predictor combinations: (A) putaminal ADC and MCP ADC; (B) putaminal χ_para_ and putaminal ADC; (C) putaminal χ_para_ and MCP ADC; and (D) putaminal χ_para_, putaminal ADC, and MCP ADC. The putaminal χ_para_–MCP ADC model in panel C, selected for primary presentation based on the full-cohort voxel-wise and ROI findings, yielded a LOOCV AUC of 0.927. The tables show the unstandardized coefficients estimated from the corresponding logistic-regression models fitted to the full cohort. Predictors were entered without standardization or rescaling, with χ_para_ expressed in ppm and ADC in mm²/s. MSA-P was coded as 1 and PD as 0. After all leave-one-out predictions had been generated, decision thresholds were selected using the Youden index applied to the pooled predictions. The thresholds were 0.40231, 0.34251, 0.50903, and 0.47018 for panels A–D, respectively.

**Supplementary Figure 6. Native- and standard-space χ_para_ maps**

Native-space χ_para_ maps of age- and sex-matched MSA-P and PD patients (top), corresponding maps registered to standard space (middle), and registered maps with deep-gray-matter atlas masks overlaid (bottom) are shown for representative participants with PD and MSA-P. Yellow arrows indicate the posterolateral putamen. The colored contours indicate the caudate nucleus, putamen, and globus pallidus atlas masks. The posterolateral putaminal χ_para_ alteration in the MSA-P participant is visible in native space and remains observable after registration. All images are displayed using the same χ_para_ range of 0–200 ppb.

**Supplementary Figure 7. Registration-quality examples across the distribution of principal ROI values.**

Registered χ_para_ maps with bilateral putamen atlas overlays (A) and registered ADC maps with bilateral MCP atlas overlays (B) are shown for participants nearest the group-specific 25th, 50th, and 75th percentiles of the corresponding bilateral ROI mean values. Red contours indicate the ROI boundaries. All images within each modality use the same display range: 0–200 ppb for χ_para_ and 0.5–2.0 × 10⁻³ mm²/s for ADC. Participants were selected according to their ROI values rather than image appearance.

**Supplementary Figure 8. Idiopathic Parkinson’s disease (PD) case with ADC false-positive**

A 76-year-old female patient with PD (disease duration 29 months). **(A)** SWI shows normal putaminal signal**. (B)** ADC map demonstrates increased diffusivity in the lateral aspect of the right putamen, which represents a false-positive finding. **(C)** This corresponds to perivascular spaces visible on T2-weighted imaging.
